# Comparison of Points of Departure for Health Risk Assessment Based on High-Throughput Screening Data

**DOI:** 10.1289/EHP408

**Published:** 2016-07-06

**Authors:** Salomon Sand, Fred Parham, Christopher J. Portier, Raymond R. Tice, Daniel Krewski

**Affiliations:** 1 Department of Risk Benefit Assessment, National Food Agency, Uppsala, Sweden; 2 McLaughlin Centre for Population Health Risk Assessment, University of Ottawa, Ottawa, Ontario, Canada; 3 Division of the National Toxicology Program, National Institute of Environmental Health Sciences, Research Triangle Park, North Carolina, USA; 4 Department of Toxicogenomics, Maastricht University, Maastricht, Netherlands; 5 Risk Sciences International, Ottawa, Ontario, Canada

## Abstract

**Background:**

The National Research Council’s vision for toxicity testing in the 21st century anticipates that points of departure (PODs) for establishing human exposure guidelines in future risk assessments will increasingly be based on *in vitro* high-throughput screening (HTS) data.

**Objectives:**

The aim of this study was to compare different PODs for HTS data. Specifically, benchmark doses (BMDs) were compared to the signal-to-noise crossover dose (SNCD), which has been suggested as the lowest dose applicable as a POD.

**Methods:**

Hill models were fit to > 10,000 *in vitro* concentration–response curves, obtained for > 1,400 chemicals tested as part of the U.S. Tox21 Phase I effort. BMDs and lower confidence limits on the BMDs (BMDLs) corresponding to extra effects (i.e., changes in response relative to the maximum response) of 5%, 10%, 20%, 30%, and 40% were estimated for > 8,000 curves, along with BMDs and BMDLs corresponding to additional effects (i.e., absolute changes in response) of 5%, 10%, 15%, 20%, and 25%. The SNCD, defined as the dose where the ratio between the additional effect and the difference between the upper and lower bounds of the two-sided 90% confidence interval on absolute effect was 1, 0.67, and 0.5, respectively, was also calculated and compared with the BMDLs.

**Results:**

The BMDL_40_, BMDL_25_, and BMDL_18_, defined in terms of extra effect, corresponded to the SNCD_1.0_, SNCD_0.67_, and SNCD_0.5_, respectively, at the median. Similarly, the BMDL_25_, BMDL_17_, and BMDL_13_, defined in terms of additional effect, corresponded to the SNCD_1.0_, SNCD_0.67_, and SNCD_0.5_, respectively, at the median.

**Conclusions:**

The SNCD may serve as a reference level that guides the determination of standardized BMDs for risk assessment based on HTS concentration–response data. The SNCD may also have application as a POD for low-dose extrapolation.

**Citation:**

Sand S, Parham F, Portier CJ, Tice RR, Krewski D. 2017. Comparison of points of departure for health risk assessment based on high-throughput screening data. Environ Health Perspect 125:623–633; http://dx.doi.org/10.1289/EHP408

## Introduction

The establishment of health-based guidance values is a key outcome of assessing the risk of chemical agents. The determination of such values includes the derivation of a point of departure (POD) from dose–response modeling or, more traditionally, use of the no-observed-adverse-effect-level (NOAEL). Dose–response modeling approaches, specifically the benchmark dose (BMD) method, are generally regarded by many international health organizations as the method of choice for derivation of the POD [Davis et al. 2011; European Food Safety Authority (EFSA) 2009].

For nongenotoxic agents, uncertainty factors accounting for inter- and intra-species differences are applied to the POD derived from the critical effect observed in animals or humans (Dourson et al. 1996). This results in a health-based guidance value, such as a tolerable daily intake (TDI), an acceptable daily intake (ADI), a reference dose (RfD), or a reference concentration (RfC). Although the exact formulation of the TDI/ADI [World Health Organization/International Programme on Chemical Safety (WHO/IPCS) 2004] differs to some extent from that for the RfD/RfC, these quantities are derived in essentially the same manner and can thus be interpreted similarly. The TDI/ADI/RfD is generally set for dietary exposure, whereas the RfC is generally set for occupational exposures occurring via inhalation; an extensive discussion of occupational exposure limits can be found in Deveau et al. (2015).

In the case of a genotoxic agent, the U.S. EPA risk-assessment guidelines recommend low-dose linear extrapolation when *a*) there are data to indicate that the dose–response curve has a linear component below the POD, or *b*) as a default for a tumor site where the mode of action is not established (U.S. EPA 2005). Linear extrapolation to low doses permits upper-bound estimates of risk at exposure levels of interest as well as estimation of “risk-specific doses” associated with specific (upper-bound) risk levels; the typical U.S. EPA target range for risk management is a 1/1,000,000 to 1/10,000 increased lifetime risk (U.S. EPA 2005). In contrast, both the European Food Safety Authority (EFSA) and the Joint FAO (Food and Agriculture Organization of the United Nations)/WHO Expert Committee on Food Additives (JECFA) have recommended a margin of exposure (MOE) approach rather than low-dose linear extrapolation for evaluating compounds that are both genotoxic and carcinogenic. EFSA and the JECFA considered that the MOE had the potential to help risk managers to distinguish between large, intermediate, and low health concerns, and thus to provide guidance for setting priorities for risk management actions (Barlow et al. 2006). The MOE is also cited in the U.S. EPA guidelines but is positioned as a quantity that provides an indication of the extent of extrapolation of risk estimates from the observed data to the exposure levels of interest in practice (U.S. EPA 2005).

Traditional approaches to risk assessment, including the establishment of health-based guidance values based on the results of mammalian toxicology tests, have been challenged by the U.S. National Research Council (NRC) in its report, *Toxicity Testing in the 21st Century: A Vision and a Strategy* (NRC 2007). This report envisions that future toxicity tests will be conducted largely in human cells or cell lines *in vitro* by evaluating cellular responses in a suite of toxicity pathway assays using high-throughput tests. Risk assessments would be performed based on the results of such tests, and the equivalents of today’s health-based guidance values would aim, according to the NRC, at representing dose levels that avoid significant perturbations of the toxicity pathways in exposed human populations. *In vitro* to *in vivo* extrapolations would rely on pharmacokinetic models to predict human blood and tissue concentrations under specific exposure conditions (Andersen and Krewski 2009; Krewski et al. 2009, 2011; NRC 2007). The NRC vision for the future of toxicity testing has recently been incorporated into the U.S. EPA’s framework for the next generation of risk science (Krewski et al. 2014).

In line with this vision, Judson et al. (2011) presented a framework for estimating the human dose at which a chemical significantly alters biological pathways *in vivo*, making use of *in vitro* assay data and an *in vitro*–derived pharmacokinetic model, along with information on population variability and uncertainty. Judson et al. (2011) calculated a “biological pathway altering dose” (BPAD), which they regarded as conceptually analogous to current risk-assessment metrics in that it combines dose–response data with analysis of uncertainty and population variability to arrive at conservative human exposure limits. Further discussion is needed on how a “biological significant perturbation,” and hence the BPAD, or related metric, should be defined. At a general level, in response to the NRC (2007), Crump et al. (2010) considered four possible definitions that were all regarded to incorporate the notion of an exposure threshold for apical response. At a more detailed level, this problem formulation may also concern the technical definition of the POD from a statistical standpoint, which is the focus of the present paper.

Historically, several approaches have been presented in the scientific literature on how to define the BMD and its lower confidence limit (BMDL) (Crump 1984; Murrell et al. 1998; Sand et al. 2006, 2008, 2011; Slob and Pieters 1998). In their opinion on the BMD, EFSA recommended a default setting for implementation of the BMD approach: in the case of quantal data, they recommended that the BMD by default be defined as the dose corresponding to an extra risk of 10%, and for continuous (experimental) data, they recommended that the BMD by default be defined as corresponding to a 5% change in response relative to the mean background response (EFSA 2009). The guidance provided by the U.S. EPA is similar to that issued by EFSA for quantal data, but the default approaches for continuous data differ between the two agencies (Davis et al. 2011).

Sand et al. (2011) introduced the concept of the signal-to-noise crossover dose (SNCD) as an objective approach to determine the lowest dose applicable as a POD, such that its corresponding effect is not overwhelmed by biological noise or uncertainty in the data. Specifically, the SNCD is defined as the dose at which the ratio between the additional effect (the “signal”) and the difference between the upper and lower bounds of the two-sided 90% confidence interval on absolute effect (the “noise”) correspond to some critical value (critical signal-to-noise ratios of 1, 0.67, and 0.5 are used in the present study). Sand et al. (2011) compared BMDLs and NOAELs to the SNCD, using values derived from fitting concentration–response data from the U.S. National Toxicology Program (NTP) carcinogenesis bioassay database. The NTP cancer studies represent one of the types of toxicity data that are currently used as a basis for risk assessment. Motivated by the anticipated shift towards the use of *in vitro* rather than whole-animal bioassay data as the basis for risk assessment, the present study extended the comparison of different BMDLs with the SNCD to the case of high-throughput *in vitro* screening data. Using the SNCD as a statistical reference point, this study aimed to provide insights into how low response levels in general may be associated with BMDs based on HTS data; the role of the SNCD as a starting point for low-dose extrapolation is also discussed. The analysis performed was based on > 10,000 *in vitro* concentration–response curves generated on > 1,400 compounds as part of the U.S. Tox21 Phase I effort (Tice et al. 2013).

## Materials and Methods

### Dose–Response Data

The Tox21 program (Tice et al. 2013) is a collaboration between U.S. federal health research agencies for the purpose of developing and applying new methods for chemical toxicity testing. Phase I of the Tox21 program tested ~2,800 chemicals, half of which were chosen by the NTP and half of which were chosen by the U.S. EPA. The chemicals were tested in > 50 high-throughput screening assays. Data from the Tox21 Phase I assays consist of 14- or 15- point concentration–response curves. Analysis of compound concentration–response data was performed as described (Inglese et al. 2006). Briefly, raw 1,536-well plate reads for each titration point were first normalized relative to the assay-specific positive control compound (100%) and dimethyl sulforxide (DMSO)-only wells (basal, 0%) on the same 1,536-well plate and then were corrected by applying a pattern correction algorithm using the compound-free 1,536-well control plates (i.e., DMSO-only plates) at the beginning and end of the compound plate stack.

### Data Selection

The assays in Phase I of Tox21 include several types of end points (Tice et al. 2013). This analysis includes three groups of assays: cytotoxicity assays, nuclear receptor assays, and assays for stress response pathways. Data sets included in this analysis are listed in Table 1. Most of these data are available in the PubChem BioAssay database (Wang et al. 2012). Each data set represents one run of an assay on one set of chemicals (U.S. EPA or NTP chemicals). Some assays were run more than once on the same chemical, or in different cell lines, or with multiple end points; those are listed as separate data sets in the table. The analysis included 47 nuclear receptor assay data sets, 23 cytotoxicity assay data sets, and 12 stress response assay data sets.

**Table 1:** Data sets used in the analysis.

Assay	PubChem BioAssay ID (AID)	Chemical source	Number of concentration–response curves in Classes 1 and 2^*a*^
Nuclear receptor assays
Human androgen receptor agonist	588515	EPA	114
Human androgen receptor antagonist	588516	EPA	289
Human estrogen α receptor agonist	588514	EPA	230
Human estrogen α receptor antagonist		EPA	429
Human farnesoid X receptor agonist	588527	EPA	20
Human farnesoid X receptor antagonist	588526	EPA	199
Human glucocorticoid receptor agonist	588532	EPA	15
Human glucocorticoid receptor antagonist	588533	EPA	154
Human peroxisome proliferator-activated receptor γ agonist	588536	EPA	181
Human peroxisome proliferator-activated receptor γ antagonist	588537	EPA	206
Human peroxisome proliferator-activated receptor δ agonist	588534	EPA	106
Human peroxisome proliferator-activated receptor δ antagonist	588535	EPA	159
Human retinoid X receptor agonist	588544	EPA	337
Human retinoid X receptor antagonist	588546	EPA	245
Human thyroid receptor agonist	588545	EPA	41
Human thyroid receptor antagonist	588547	EPA	98
Human vitamin D receptor agonist	588543	EPA	24
Human vitamin D receptor antagonist	588541	EPA	120
Human androgen receptor agonist	588515	NTP	146
Human androgen receptor antagonist	588516	NTP	367
Human aryl hydrocarbon receptor agonist	651777	NTP	86
Human estrogen α receptor agonist	588514	NTP	157
Human estrogen α receptor antagonist	588513	NTP	139
Human farnesoid X receptor agonist	588527	NTP	9
Human farnesoid X receptor antagonist	588526	NTP	211
Human glucocorticoid receptor agonist	588532	NTP	14
Human glucocorticoid receptor antagonist	588533	NTP	189
Human peroxisome proliferator-activated receptor α agonist	651778	NTP	13
Human peroxisome proliferator-activated receptor α antagonist	NA	NTP	227
Human peroxisome proliferator-activated receptor α antagonist	NA	NTP	237
Human peroxisome proliferator-activated receptor γ agonist, CHO cells	NA	NTP	16
Human peroxisome proliferator-activated receptor γ agonist, CHO cells	NA	NTP	31
Human peroxisome proliferator-activated receptor γ agonist, Hek293 cells	588536	NTP	77
Human peroxisome proliferator-activated receptor γ antagonist, Hek293 cells	588537	NTP	232
Human peroxisome proliferator-activated receptor δ agonist	588534	NTP	110
Human peroxisome proliferator-activated receptor δ antagonist	588535	NTP	245
Human pregnane X receptor agonist	720659	NTP	192
Human retinoid X receptor agonist	588544	NTP	177
Human retinoid X receptor antagonist	588546	NTP	97
Human thyroid receptor agonist	588545	NTP	89
Human thyroid receptor antagonist	588547	NTP	67
Human vitamin D receptor agonist	588543	NTP	16
Human vitamin D receptor antagonist	588541	NTP	94
Rat pregnane X receptor agonist	651751	NTP	153
Cytotoxicity assays
Viability in 3T3 cells	NA	NTP	236
Viability in BJ cells	421	NTP	80
Viability in endotoxin assay	NA	NTP	334
Viability in glucocorticoid receptor assay	NA	NTP	111
Viability in H-4-II-E cells	543	NTP	231
Viability in Hek293 cells	131	NTP	131
Viability in HeLa cells in the antioxidant response element assay	NA	NTP	111
Viability in HepG2 cells in the antioxidant response element assay	720653	NTP	62
Viability in HepG2 cells	433	NTP	156
Viability in HepG2 cells	NA	NTP	189
Viability in HepG2 cells	NA	NTP	173
Viability in HUVEC cells	542	NTP	110
Viability in Jurkat cells	426	NTP	213
Viability in mesangial cells	546	NTP	108
Viability in mesangial cells	NA	NTP	51
Viability in MRC-5 cells	434	NTP	73
Viability in N2a cells	540	NTP	202
Viability in nuclear factor κB assay	NA	NTP	27
Viability in p53 assay	743292	NTP	69
Viability in peroxisome proliferator-activated receptor α assay	NA	NTP	95
Viability in rat renal proximal tubule cells	545	NTP	159
Viability in SH-SY5Y cells	544	NTP	244
Viability in SK-N-SH cells	435	NTP	126
Stress response assays
Antioxidant response element, beta-lactamase reporter	651741	NTP	583
Antioxidant response element, luciferase reporter	720636	NTP	192
Cyclic AMP response element agonist	NA	NTP	162
Cyclic AMP response element antagonist	NA	NTP	139
Endoplasmic reticulum stress response element	NA	NTP	51
Heat shock protein, luciferase reporter	NA	NTP	7
Heat shock protein, luciferase reporter	NA	NTP	31
Heat shock protein, beta-lactamase reporter	NA	NTP	24
Hypoxia inducible factor 1	2120	NTP	73
Nuclear factor κB agonist	651749	NTP	26
Nuclear factor κB antagonist	NA	NTP	231
p53 gene	651743	NTP	72
Notes: EPA, U.S. Environmental Protection Agency; NA, not available on PubChem; NTP, National Toxicology Program. ^***a***^Each concentration–response curve has a curve classification, based on the fit of a Hill equation to the curve (Xia et al. 2011; Huang et al. 2011). For this analysis, only curves in classes 1 and 2 (“complete response curve” and “incomplete curve,” respectively) were used because the other curve classes indicate the lack of a concentration response or show significant activity only at the highest concentration and are therefore problematic for the purpose of fitting a sigmoidal (four-parameter) model such as the Hill model.			

In addition to the concentration and response data, each concentration–response curve has a curve classification based on the fit of a Hill equation to the curve (Xia et al. 2011; Huang et al. 2011). There have been two slightly different systems of curve classification. When the more recent curve classification (Huang et al. 2011) became available, it was used; otherwise, the classification from the older system was used (Xia et al. 2011). For this analysis, only curves in classes 1 and 2 (“complete response curve” and “incomplete curve,” respectively) were used because the other curve classes indicate the lack of a concentration response or show significant activity only at the highest concentration and are therefore problematic for the purpose of fitting a sigmoidal (four parameter) model, such as the Hill model. Thus, the present work was limited to address POD derivation for concentration–response curves that are fairly well characterized, as in the previous study using this method (Sand et al. 2011). The assays include replicated data for some of the study chemicals. The present analysis in this paper does not take replication into account, that is to say, replicates were considered as separate concentration–response curves; however, an extended analysis focusing on NTP duplicates was also performed. The number of concentration–response curves used from each data set is given in Table 1. The data normalization and curve classification process includes outlier determination. Outlier points, as specified in the data obtained from Tox21, were not included in the fitting of the Hill function to the data.

### Dose–Response Modeling and Estimation of PODs

Dose–response modeling was performed using the Hill model fit to the data by maximum likelihood, with a parametric bootstrap approach for obtaining confidence limits on the PODs derived from the fitted model. The 11,240 concentration–response curves included as a starting point in the analysis were modeled using an automated protocol developed in Matlab (The MathWorks, Inc.). The details associated with the model-fitting approach and POD estimation can be found in “Concentration–response modeling and estimation of PODs” in the Supplemental Material. The quantities described below were estimated for each curve.

The BMD, with a two-sided 90% confidence interval, corresponding to extra effects of 5%, 10%, 20%, 30%, and 40%. The extra effect is defined as a percent change in response relative to the estimated range of response. A subscript “e” is used to denote these BMDs (e.g., BMD_e_, BMDL_e_, BMD_10e_, BMDL_10e_).
The BMD, with a two-sided 90% confidence interval, corresponding to additional effects of 5%, 10%, 15%, 20%, and 25%. The additional effect is defined as an absolute change in response compared to the estimated background response. A subscript “a” is used to denote these BMDs (e.g., BMD_a_, BMDL_a_, BMD_10a_, BMDL_10a_).
The SNCD corresponding to signal-to-noise ratios of 1.0, 0.67, and 0.5, denoted by SNCD_1.0_, SNCD_0.67_, and SNCD_0.5_, respectively. The point estimate, as well as the upper 95th confidence bound, for the effect (under both the additional and extra effect definitions) at concentrations corresponding to each of the three SNCDs was also derived.

**Figure 1:**
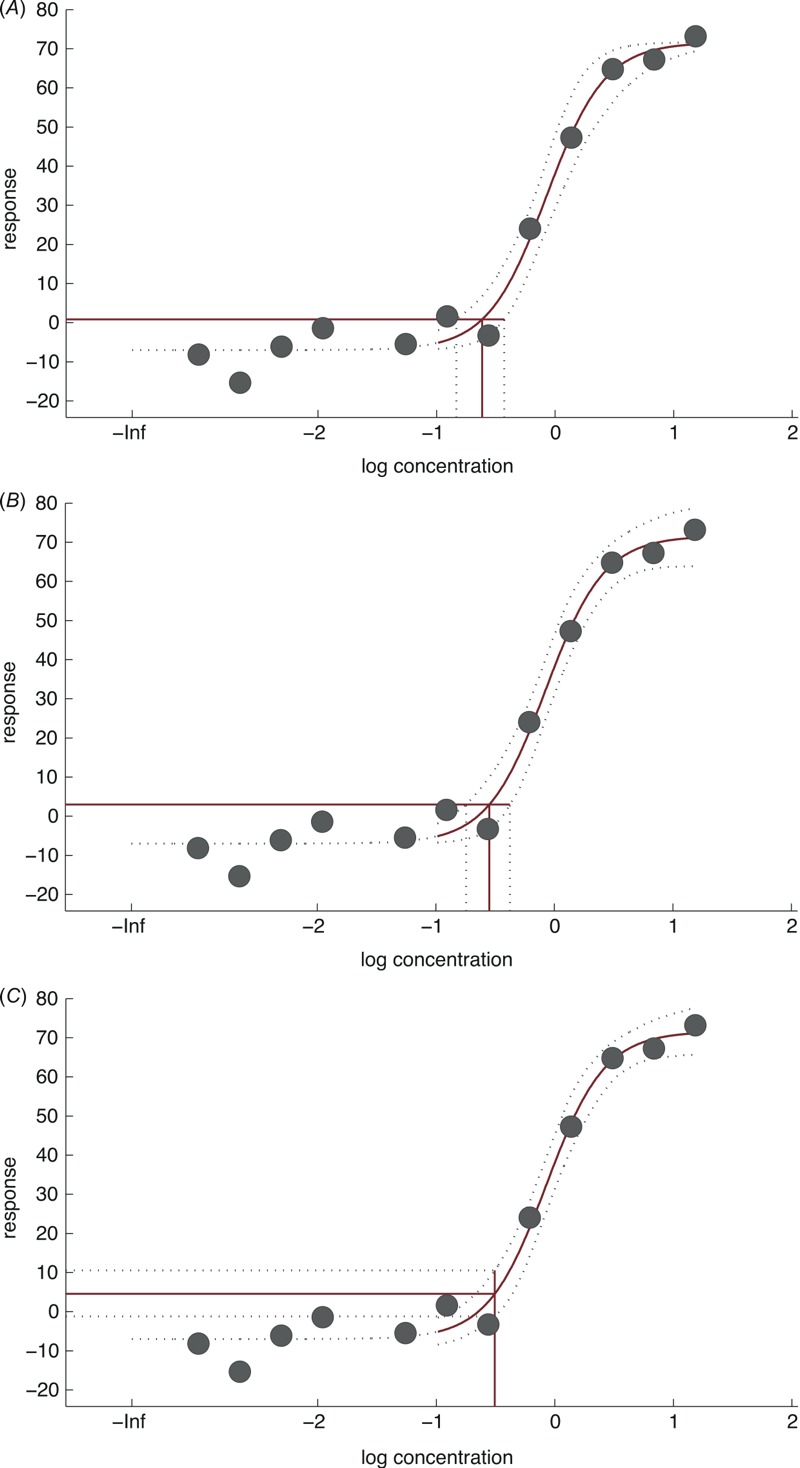
Illustration of the three types of point-of-departure (POD) approaches considered in the study. Nuclear receptor assay concentration response data on pimozide is used as an example (solid circles). The Hill model has been fitted to the data: in all three cases, the solid curves that describe the mean response are the same, but the two-sided 90% confidence intervals around the mean response (the dotted curves) depend on the POD approach considered. (*A*) The benchmark dose (BMD) associated with a 10% extra effect (BMD_10e_) is 0.24 units (solid red vertical line), and the lower 5th and upper 95th confidence limits (vertical dotted lines) are 0.15 (BMDL_10e_) and 0.37 units, respectively. (*B*) The BMD associated with a 10% additional effect (BMD_10a_) is 0.28 units (solid red vertical line), and the lower 5th and upper 95th confidence limits (vertical dotted lines) are 0.18 (BMDL_10a_) and 0.42 units, respectively. (*C*) The SNCD_1.0_ associated with a signal-to-noise ratio (SNR) of 1.0 is 0.31 units (solid red vertical line). The difference between the lower and upper bounds on absolute effect at the SNCD is ≈ 10.4 – (–1.2) = 11.6 (difference between the horizontal dotted lines). Because the SNR is 1.0, this approximates to the point estimate of additional effect at the signal-to-noise crossover dose (SNCD), which is ≈ 4.6 – (–7.0) = 11.6 (difference between the horizontal solid line and the background response according to the fitted model). In this example, SNCD_1.0_ is approximately twice the size of the BMDLs.

The three types of POD approaches (BMD_e_, BMD_a_, and SNCD) are illustrated in Figure 1. Additionally, a discussion of the BMD and SNCD definitions, including why the applied BMD definitions were preferred over the definition suggested for continuous data by EFSA (2009), is provided in “Definition of the SNCD and the BMD” in the Supplemental Material.

### Comparison of PODs

BMDLs were compared to the SNCD (specifically, SNCD_1.0_, SNCD_0.67_, and SNCD_0.5_). These comparisons were based on curves for which all estimated BMDs and SNCDs (in total, 10 BMDs and 3 SNCDs) were within the experimental concentration range (*n* = 8,961). In addition, results associated with nonsignificant concentration–response curves (*n* = 192) and curves for which the estimated maximum response was > 150 or < –150 (*n* = 313 additional curves) were excluded. These combined criteria reduced the 11,240 curves by 25% to 8,456 curves for inclusion in the present study. As noted previously, details of the model-fitting approach and POD estimation can be found in “Concentration–response modeling and estimation of PODs” in the Supplemental Material.

## Results

### BMDLs Based on Extra Effect versus the SNCD

**Figure 2:**
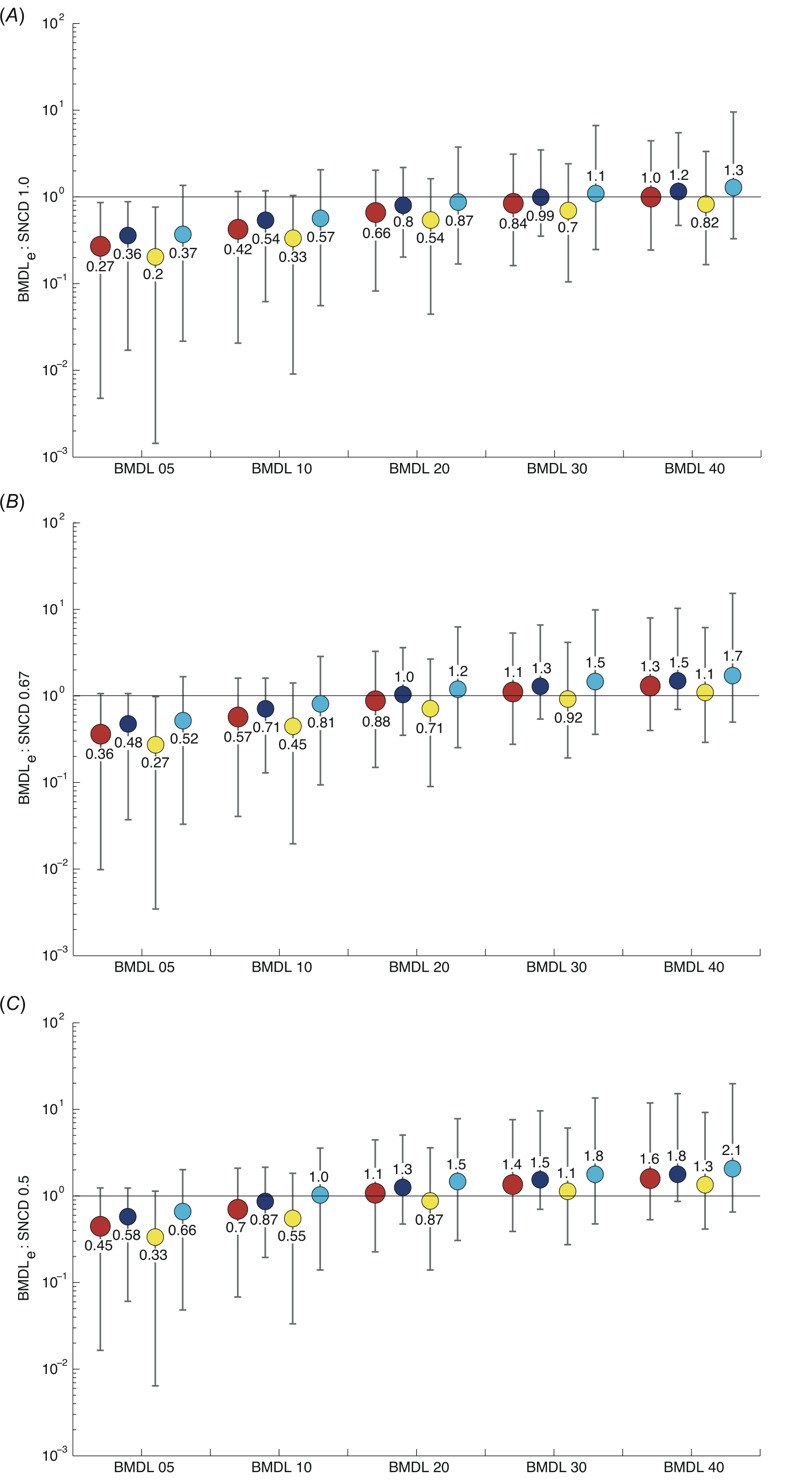
Ratios of the BMDL_e_ to the SNCD with BMDLs defined in terms of extra effects of 5%, 10%, 20%, 30%, and 40%. Ratios are given in terms of medians (solid circles) and intervals describing the lower 5th and upper 95th percentiles, based on different stratifications of the data. Red (large) circles correspond to results based on all selected curves (*n *= 8,456); blue circles correspond to results based on cytotoxicity assays (*n *= 3,130); yellow circles correspond to results based on nuclear receptor assays (*n *= 4,603); and cyan circles are results based on stress response assays (*n *= 723). (*A*) Ratios of the BMDL_e_ to the SNCD_1.0_. (*B*) Ratios of the BMDL_e_ to the SNCD_0.67_. (*C*) Ratios of the BMDL_e_ to the SNCD_0.5_. BMDL, lower confidence limit of the benchmark dose; SNCD, signal-to-noise crossover dose.

Considering all curves selected for inclusion (*n* = 8,456), the BMDL_40e_ calibrated to the SNCD_1.0_ at the median (Figure 2A). A concentration between the BMDL_20e_ and the BMDL_30e_ corresponded to the SNCD_1.0_ for stress response assays; the BMDL_30e_ calibrated to the SNCD_1.0_ for cytotoxicity assays; and all BMDLs were below the SNCD_1.0_ at the median for nuclear receptor assays (Figure 2A).

**Figure 3:**
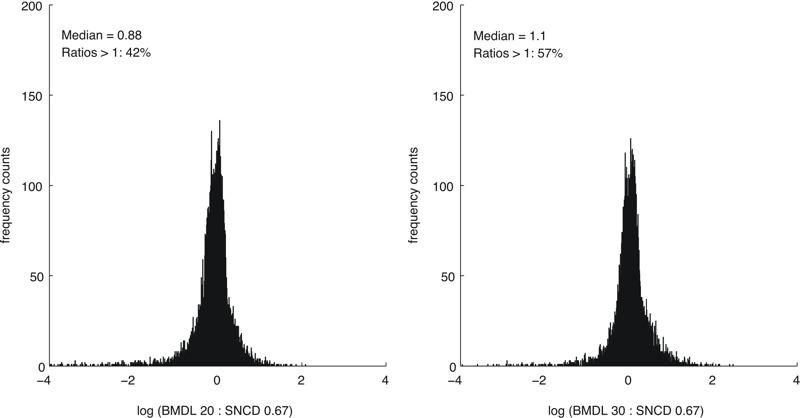
Histograms for the ratios BMDL_e_:SNCD_0.67_ (BMDLs are based on extra effect) with medians closest to 1 based on all included curves (*n *= 8,456). BMDL, lower confidence limit of the benchmark dose; SNCD, signal-to-noise crossover dose.

A concentration level between the BMDL_20e_ and the BMDL_30e_ corresponded to the SNCD_0.67_, at the median, across all *n* = 8,456 curves (Figure 2B). A concentration between the BMDL_10e_ and the BMDL_20e_ corresponded to the SNCD_0.67_ for stress response assays; the BMDL_20e_ calibrated to the SNCD_0.67_ for cytotoxicity assays; and a concentration between the BMDL_30e_ and the BMDL_40e_ corresponded to the SNCD_0.67_ for nuclear receptor assays (Figure 2B). Histograms for the ratios BMDL:SNCD_0.67_ with medians closest to 1 are shown in Figure 3 (considering all *n* = 8,456 curves).

At the median, the BMDL_20e_ was closest to the SNCD_0.5_ when all 8,456 curves were considered (Figure 2C). The BMDL_10e_ calibrated to the SNCD_0.5_ for stress response assays; the BMDL_10e_ was closest to the SNCD_0.5_ for cytotoxicity assays; and a concentration between the BMDL_20e_ and the BMDL_30e_ corresponded to the SNCD_0.5_ for nuclear receptor assays (Figure 2C).

### BMDLs Based on Additional Effect versus the SNCD

**Figure 4:**
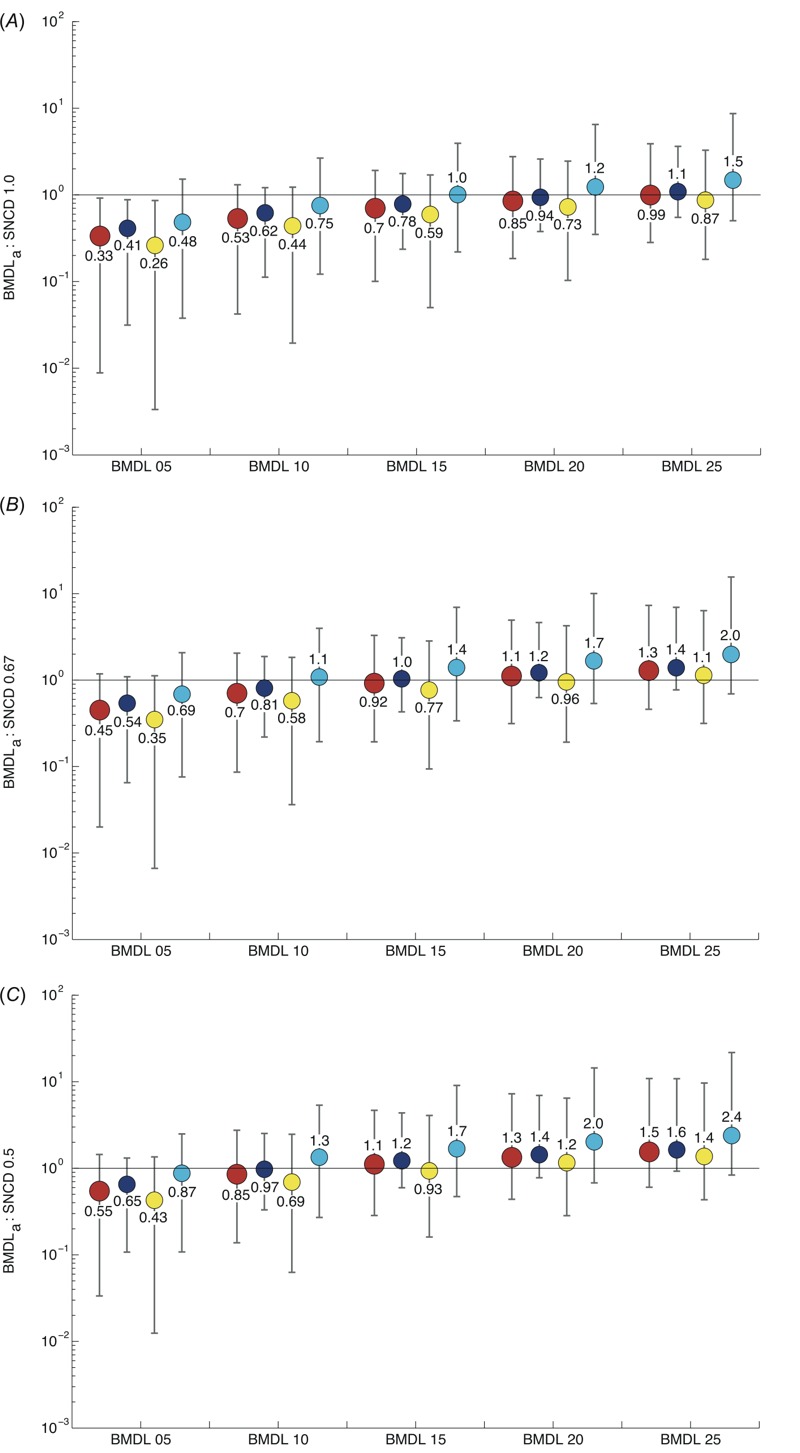
Ratios of the BMDL_a_ to the SNCD with BMDLs defined in terms of additional effects of 5%, 10%, 15%, 20%, and 25%. Ratios are given in terms of medians (solid circles) and intervals describing the lower 5th and upper 95th percentiles, based on different stratifications of the data. Red (large) circles correspond to results based on all selected curves (*n *= 8,456); blue circles correspond to results based on cytotoxicity assays (*n *= 3,130); yellow circles correspond to results based on nuclear receptor assays (*n *= 4,603); and cyan circles are results based on stress response assays (*n *= 723). (*A*) Ratios of the BMDL_a_ to the SNCD_1.0_. (*B*) Ratios of the BMDL_a_ to the SNCD_0.67_. (*C*) Ratios of the BMDL_a_ to the SNCD_0.5_. BMDL, lower confidence limit of the benchmark dose; SNCD, signal-to-noise crossover dose.

Considering all included curves (*n* = 8,456), the BMDL_25a_ calibrated to the SNCD_1.0_ at the median (Figure 4A). The BMDL_15a_ calibrated to the SNCD_1.0_ for stress response assays; a concentration between the BMDL_20a_ and the BMDL_25a_ corresponded to the SNCD_1.0_ for cytotoxicity assays; and all BMDLs were below the SNCD_1.0_ at the median for nuclear receptor assays (Figure 4A).

**Figure 5:**
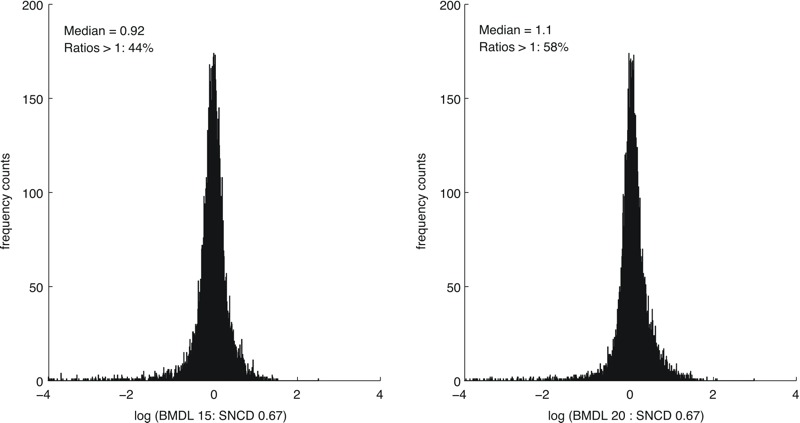
Histograms of the ratios BMDL_a_:SNCD_0.67_ (BMDLs are based on additional effect) with medians closest to 1 based on all included curves (*n *= 8,456). BMDL, lower confidence limit of the benchmark dose; SNCD, signal-to-noise crossover dose.

At the median, the SNCD_0.67_ lay between the BMDL_15a_ and the BMDL_20a_ for all curves (*n* = 8,456) (Figure 4B). The BMDL_10a_ was closest to the SNCD_0.67_ for stress response assays; the BMDL_15a_ calibrated to the SNCD_0.67_ for cytotoxicity assays; and a concentration between the BMDL_20a_ and the BMDL_25a_ corresponded to the SNCD_0.67_ for nuclear receptor assays (Figure 4B). Histograms for the ratios BMD:SNCD_0.67_ with medians closest to 1 are shown in Figure 5 (considering all *n* = 8,456 curves).

At the median, the SNCD_0.5_ lay between the BMDL_10a_ and the BMDL_15a_ when all curves (*n* = 8,456) were considered (Figure 4C). The BMDL_05a_ was closest to the SNCD_0.5_ for stress response assays; the BMDL_10_ approximated to the SNCD_0.5_ for cytotoxicity assays; and a concentration between the BMDL_15a_ and the BMDL_20a_ corresponded to the SNCD_0.5_ for nuclear receptor assays (Figure 4C).

### Effect at the SNCD

**Figure 6:**
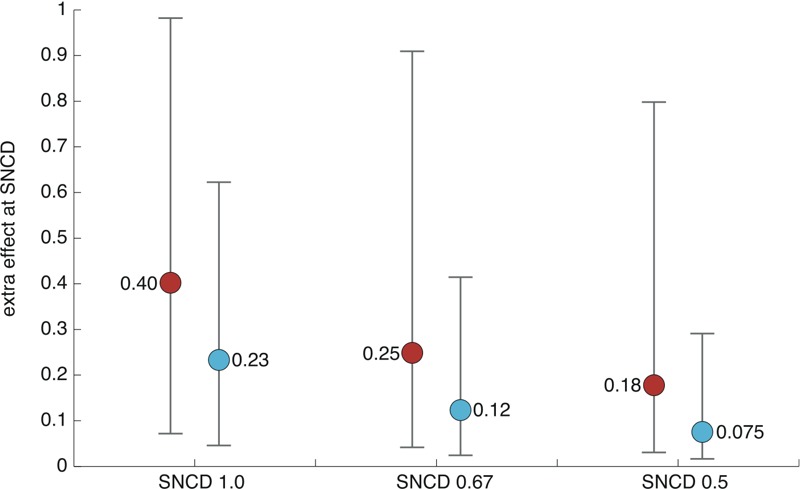
Extra effect at the SNCD. Medians (solid circles) and intervals describing the lower 5th and upper 95th percentiles are shown based on all included curves (*n *= 8,456). Red circles correspond to the upper bound of the effect, and cyan circles correspond to the point estimate of the effect. SNCD, signal-to-noise crossover dose.

**Figure 7:**
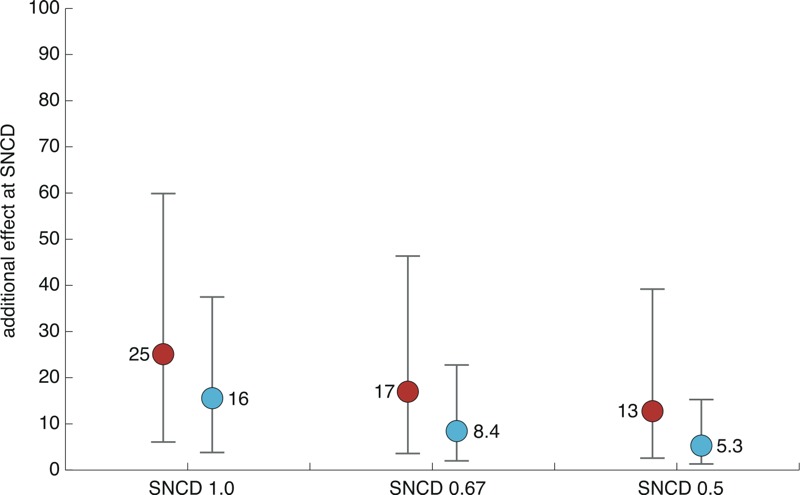
Additional effect at the SNCD. Medians (solid circles) and intervals describing the lower 5th and upper 95th percentiles are based on all included curves (*n *= 8,456). Red circles correspond to the upper bound of the effect, and cyan circles correspond to the point estimate of the effect. BMDL, lower confidence limit of the benchmark dose; SNCD, signal-to-noise crossover dose.

Figures 6 and 7 show the medians, as well as the lower 5th and upper 95th percentiles, for the extra and additional effects at the SNCD, respectively, using all included curves (*n* = 8,456) as the basis. These results indicate that the SNCD_1.0_, SNCD_0.67_, and SNCD_0.5_ corresponded to a median upper bound on the extra effect of 40% (corresponding to the BMDL_40e_), 25% (corresponding to a concentration between BMDL_20e_ and BMDL_30e_), and 18% (corresponding approximately to the BMDL_20e_), respectively (Figure 6). Similar results in Figure 7 show that the SNCD_1.0_, SNCD_0.67_, and SNCD_0.5_ corresponded to a median upper bound of the additional effect of 25% (corresponding to the BMDL_25a_), 17% (corresponding to a concentration between the BMDL_15a_ and the BMDL_20a_), and 13% (corresponding to a concentration between the BMDL_10a_ and the BMDL_15a_), respectively. The results illustrated in Figures 6 and 7 are consistent with those presented in Figures 2–5.

### Analysis of NTP Duplicates

Chemicals tested in duplicate on the NTP assay plates were analyzed separately to investigate the stability of estimated quantities across duplicates, as well as the result of merging duplicates. Considering curves in classes 1 and 2 (“complete response curve” and “incomplete curve,” respectively), on which the overall analysis is based, 320 duplicates were identified (i.e., 640 individual curves). At the median, the BMDL differed between these duplicates by a factor of 1.6–2.2 for BMDLs defined in terms of extra effect and a factor of 1.6–2.0 for BMDLs defined in terms of additional effect: the differences decreased with increasing BMR (Table 2). At the median, the SNCD differed between duplicates by a factor of 1.7–1.8, depending on the SNR (Table 2). It may be noted that the upper 95th percentile of the BMDL ratio across duplicates was very high at low BMRs, ranging between 100 and 600 depending on the BMR. For other BMDLs, the upper 95th percentile of the ratio of difference between duplicates was in the range of 20- to 40-fold for BMDLs defined in terms of extra effect and 30- to 50-fold for BMDLs defined in terms of additional effect. For the SNCD, the upper 95th percentile of the ratio of difference between duplicates was in the range of 30-fold.

**Table 2:** Comparison of BMDLs and SNCDs for NTP duplicates.

Type of comparison	Quantity	Median	5th percentile	95th percentile	*X*^*e*^
BMDL ratio between duplicates (extra effect)^*a*^	BMDL_05e_	2.2	1.0	625	—
	BMDL_10e_	1.9	1.0	140	—
	BMDL_20e_	1.7	1.0	43	—
	BMDL_30e_	1.6	1.0	26	—
	BMDL_40e_	1.6	1.0	17	—
BMDL ratio between duplicates (additional effect)^*b*^	BMDL_05a_	2.0	1.0	455	—
	BMDL_10a_	1.7	1.0	104	—
	BMDL_15a_	1.6	1.0	51	—
	BMDL_20a_	1.6	1.0	32	—
	BMDL_25a_	1.6	1.0	29	—
SNCD ratio between duplicates^*c*^	SNCD_1.0_	1.7	1.0	29	—
	SNCD_0.67_	1.7	1.0	28	—
	SNCD_0.5_	1.8	1.0	35	—
SNCD_duplicate GM_:SNCD_merged_^*d*^	SNCD_1.0_	1.0	0.45	3.1	0.58
	SNCD_0.67_	1.1	0.47	3.0	0.62
	SNCD_0.5_	1.1	0.44	3.1	0.63
Notes: BMDL, lower confidence limit of the benchmark dose; GM, geometric mean; NTP, National Toxicology Program; POD, point of departure; SNCD, signal-to-noise crossover dose. The analysis is based on 307 duplicates (614 individual curves). There are a total of 320 NTP duplicates with curves in classes 1 and 2; that is, 320–307 = 13 curves have been excluded from this analysis because they did not show a concentration–response trend according to criteria described in “Concentration–response modeling and estimation of PODs” in the Supplemental Material. The BMDL ratios have been calculated such that they are always > 1 (max value/min value). ^***a***^Ratio of extra effect BMDLs between duplicates. ^***b***^Ratio of additional effect BMDLs between duplicates. ^***c***^Ratio of SNCDs between duplicates. ^***d***^Ratio of the geometric mean of the SNCD between duplicates (SNCD_duplicate GM_) and the corresponding SNCD resulting from analysis of merged duplicates (SNCD_merged_). ^***e***^Fraction of curves for which the ratio is > 1.					

Table 2 also provides summary information for the ratio between the geometric mean of the SNCD from separate analysis of duplicates and the SNCD associated with analysis of merged duplicates. At the median, this ratio was ~1; for ~60% of the cases, the ratio was > 1 (Table 2). Overall, the SNCD associated with the analysis of merged duplicates approximated well to the geometric mean of SNCDs from separate analysis of duplicates.

In “Analysis of NTP duplicates” in the Supplemental Material, it is shown that summary results describing the effect at the SNCD for the case of separate analysis of duplicates are very similar to the corresponding results associated with the analysis of merged duplicates, and median values for the effect at the SNCD are also similar to those obtained for the whole database (Figures 6 and 7; see also Table S1).

## Discussion

In this article, we compared two points of departure—the traditional BMDL and the recently proposed SNCD—applied to > 8,000 high-throughput experimental concentration–response curves generated during Tox21 Phase I (Tice et al. 2013). The results from these comparisons showed that the BMDL_40_, BMDL_25_, and BMDL_18_, defined in terms of extra effect, correspond to the SNCD_1.0_, SNCD_0.67_, and SNCD_0.5_, respectively, at the median (Figure 6). Similarly, the BMDL_25_, BMDL_17_, and BMDL_13_, defined in terms of additional effect, correspond to the SNCD_1.0_, SNCD_0.67_, and SNCD_0.5_, respectively, at the median (Figure 7).

Separate analysis of NTP duplicates showed that the difference in BMDLs and SNCDs between duplicates was generally within a factor of 2 at the median (Table 2). However, the difference between duplicates was large for a portion of the curves, particularly for BMDLs corresponding to low BMRs (see the upper 95th percentile of the difference between duplicates in Table 2). As shown by Sand et al. (2011), the SNCD decreases with increasing sample size because larger sample size permits the detection of smaller and smaller effects. This phenomenon was, however, not observed in the analysis of the NTP duplicates, possibly because the increase in sample size obtained by merging duplicates was too small (a factor of only 2). The dependence of the SNCD or the BMDL on sample size is typically evaluated theoretically assuming that no (or only a minimal) effect in the mean response occurs: the only effect considered is the effect of more or fewer data for a curve of the same mean response. The analyses in the present paper indicated that the difference between duplicates with respect to the mean response curve appeared to be larger, by a factor in the range of 2, than the change in SNCD that was obtained by merging duplicates: the SNCD based on the analysis of merged duplicates approximated the geometric mean of the SNCD associated with separate analysis of duplicates (Table 2).

The findings in this paper depended on the study designs used in the database, which comprised 13–16 concentrations (sometimes fewer after removing outliers) with one observation at each concentration level. SNCDs corresponding to three different SNRs (1, 0.67, and 0.5) were considered. How stringent to be with regard to the selection of the critical SNR that defines the SNCD is a point for discussion even though a critical SNR = 1 may intuitively appear to be most straightforward (“signal” equals “noise”). However, even using the least-stringent criteria (in terms of level of “noise” allowed) corresponding to an SNR of 0.5, BMDLs corresponding to responses in the range of 10% or below appear to be associated with high uncertainty using the SNCD as a reference (Figures 6 and 7). Similarly, in Figures 2 and 4, it can be noted that the BMDL_10_ is generally below the SNCDs at the median. The analysis of NTP duplicates from Tox21 Phase I also indicated that at least these HTS data could be very uncertain with respect to estimation of BMDLs corresponding to BMRs of 10% or below because such quantities could differ substantially between individual duplicates (Table 2).

For the NTP cancer bioassay data analyzed by Sand et al. (2011), the BMDL_18_ and BMDL_7.3_, defined in terms of extra risk, corresponded to the SNCD_1.0_ and SNCD_0.67_, respectively, at the median. The corresponding BMDLs in the present study would be the BMDL_40_ and BMDL_25_, based on the extra-effect definition of the BMDL. There are several factors that may explain why the SNCD corresponded to higher BMDLs in the present study than those in the study by Sand et al. (2011). First, the data used in the present analysis were continuous in nature, complicating the ability to make a direct comparison between the two studies. In addition, a four-parameter model was used in the present study, whereas three- and two-parameter Hill models were used by Sand et al. (2011). The higher level of complexity of the four-parameter Hill model would be expected to result in wider confidence intervals, pushing the SNCD upwards. Furthermore, the SNCD is affected by sample size: whereas the NTP curves evaluated by Sand et al. (2011) typically included 200 observations (four dose groups, including the control, with 50 animals per group), the curves in the present analysis typically included only 13–16 observations (based on 1 observation per concentration). Moreover, a bootstrap approach was used in the present study for confidence interval estimation, whereas the profile likelihood method was used by Sand et al. (2011). In contrast to the analysis by Sand et al. (2011), the present analysis adjusted the estimate of variance (the likelihood estimator of the variance) to an unbiased estimator (see “Concentration–response modeling and estimation of PODs” in the Supplemental Material) in the process of confidence interval estimation. This adjustment increased the variance (sometimes marginal, depending on the sample size), which increased the SNCD. Additionally, for these reasons, the BMDL:SNCD ratio may be smaller under the applied bootstrap approach than under the profile likelihood method. Further analysis is needed to investigate the impact of model dependence (with respect to the mean response model) of the results associated with this analysis. The relatively large number of concentration levels (generally 13–16) will, however, constrain dose–response models such that they may not assume very different shapes (in the observable region of response). Using normalized data will tend to decrease the variance and therefore decrease the SNCD.

As an example of the use of the SNCD in a risk-assessment context, Sand et al. (2011) illustrated how an SNCD-based exposure guideline based on low-dose linear extrapolation, using the upper bound on extra risk at the SNCD as a starting point, might be calculated. The SNCD appears consistent with the definition of a POD given in the U.S. EPA (2005) cancer guidelines, which state that a POD “marks the beginning of extrapolation to lower doses.” Burgoon and Zacharewski (2008) described a POD in a way that conceptually resembles the SNCD: their POD was defined “as the point at which the upper 95% confidence limit for the vehicle response intersects the lower 95% confidence limit for the treated response based on parametric assumptions.”

The description of the SNCD and the illustration of its potential uses given by Sand et al. (2011) are statistical in nature. However, it has also been suggested that a POD derived from dose–response modeling should include a toxicological interpretation. For example, EFSA’s opinion on the BMD states that the response (benchmark response, BMR) associated with the BMD should be in the range of the data to avoid having to estimate a BMD by extrapolation. EFSA also notes that their default recommendations, which are based on calibration to the NOAEL approach, may be modified based on statistical or toxicological considerations (EFSA 2009).

Considering both statistical and biological aspects of the POD, Chiu et al. (2012) and Sand et al. (2012a) argued that the SNCD may represent a starting point for low-dose extrapolation when the upper bound on the risk (or effect) at the SNCD is greater than a “target effect level” (or BMR) established based on biological (Chiu et al. 2012; Sand et al. 2012a) or risk-management (Sand et al. 2012a) considerations. In case the SNCD is below the target effect level, the dose associated with that effect may be directly used as a POD (Chiu et al. 2012).

According to the NRC (2007) vision for the future of toxicity testing, increasing attention will be redirected towards determining exposure levels that avoid significant perturbations in toxicity pathways. Judson et al. (2011) introduced the concept of biological pathway activating dose (BPAD) and, as a starting point for the establishment of the BPAD, used the ToxCast^TM^ AC_50_ values (the concentration at 50% of maximum activity) as PODs in their illustration of the BPAD concept. AC_50_ values have also been considered in other analyses of *in vitro* data (Burgoon and Zacharewski 2008; Thomas et al. 2012; Wetmore et al. 2012). As an alternative to using the AC_50_, Sand et al. (2012b) suggested that the dose at which the slope of the S-shaped dose–response curve changes the most per unit log-dose, denoted BMD_T_, may serve as a standardized reference point in the low dose–region for *in vitro* data. The BMD_T_/BMDL_T_, which approximates the BMD_20_/BMDL_20_ using the extra effect definition under the Hill model, was introduced by Sand et al. (2006) and was suggested as a mathematical definition of a dose within a “transition dose range,” as discussed by Slikker et al. (2004). Derivation of PODs like the BMD_T_ as well as the AC_50_ requires adequate characterization of the S-shaped concentration–response curve (including the asymptotes).

As noted in “Methods,” only curves in classes 1 and 2 were considered in this work to support modeling of the full S-shaped curve. Consequently, results from this analysis are limited in this context and do not address the issue of POD derivation for concentration–response curves that are poorly characterized. Shockley (2015) concluded that to improve nonlinear parameter estimation, optimal study designs should be developed, or alternative approaches with reliable performance characteristics should be used to describe concentration–response curves; suggestions that address the latter issue have also been proposed (Hsieh et al. 2015).

It may be questioned whether derivation of PODs for *in vitro* data should involve biological, policy, or risk-management considerations regarding the effect level associated with the POD. At this point, it is unclear if avoiding “significant perturbations in toxicity pathways” would imply that some (presumably small) changes in response might be allowed with regard to the suite of critical *in vitro* end points that would be needed to be evaluated in a future risk-assessment framework (Krewski et al. 2014). Although conceptually reasonable, the determination of BMRs representing “nonadverse” response levels, or similar, for various end points is a major challenge within the current risk-assessment approach, and, if applicable, such may also be the case for *in vitro* data. An even more complex issue is determination of which changes in biological effect parameters are acceptable in the case of end points that are not adverse and are not the critical effect or its known and immediate precursor. Issues related to this point have also been discussed by Crump et al. (2010) and Sand et al. (2012b).

It is likely that derivation of PODs from *in vitro* high-throughput screening data will need to rely on standardized approaches, at least as a starting point. Because the use of *in vitro* data significantly increases the amount of concentration–response data that needs to be processed, the use of standardized modeling protocols, including standardized PODs, may be of importance, at least from a practical point of view. Wignall et al. (2014) recently discussed the use of a standardized protocol for BMD analysis that was argued to provide greater transparency and efficiency than current approaches. Their approach was illustrated for traditional animal toxicity data, but the relevance of this type of approach was also suggested to be of particular value in the case of high-throughput *in vitro* testing (Wignall et al. 2014). Thomas et al. (2013) noted that more efficient risk-assessment approaches are needed owing to the fact that the number of chemicals without toxicity reference values combined with the rate of new chemical development is overwhelming the capacity of the traditional risk-assessment approach. Interestingly, the results of their studies of comparing transcriptional BMD values for the most sensitive pathway with BMD values for the noncancer and cancer apical end points showed a high degree of correlation, suggesting that (for their studied chemicals) transcriptional perturbation did not occur at significantly lower doses than apical responses (Thomas et al. 2013).

The SNCD may provide a reference level for determining how low a standardized BMD, BMDL, or similar (potency-based) quantity may be selected. For example, in risk-assessment applications where BMDs are derived for several chemicals or end points, a default or screening POD may be chosen such that it is generally not below the SNCD. Based on the present analysis, such a screening level may be lower than the commonly used AC_50_, discussed above, because the AC_50_ (i.e., the BMDL_50_) is higher than all SNCDs at the median (Figures 6 and 7). Considering the range of SNCDs evaluated, the BMDL_20_ may be more appropriate as a standardized POD in this context (in terms of extra effect, the BMDL_20_ corresponds to a concentration between the SNCD_0.5_ and the SNCD_0.67_ at the median; in terms of additional effect, the BMDL_20_ corresponds to a concentration between SNCD_0.67_ and SNCD_1.0_ at the median) (Figures 6 and 7). As noted previously, BMDLs associated with BMRs < 10% generally appear to not be supported from a statistical point of view when using the SNCD as a reference (Figures 6 and 7). BMRs < 10% may, however, be supported for individual curves when using the SNCD as a reference.

The SNCD concept may also be used as a starting point for low-dose extrapolation in establishing exposure guidelines corresponding to a given target risk (Chiu et al. 2012; Sand et al. 2011, 2012a) using empirical models of a linear or nonlinear nature. This approach may also be viewed as the application of a curve-specific uncertainty factor to the SNCD, which depends on the risk/effect at the SNCD and the empirical extrapolation model used (Sand et al. 2011). It may be noted that, if the dose–response is sublinear, the risk estimate by the SNCD generally decreases as the sample size increases, as discussed by Sand et al. (2011). Increasing sample size lowers the SNCD, and under a linear extrapolation approach (drawing a straight line between the upper bound of risk/effect at the SNCD and the background response), the dose corresponding to a given target risk/effect then increases (less conservative) because the slope of the linear model becomes smaller. Although this approach may be appropriate for severe apical end points, the circumstances under which an approach involving low-dose extrapolation would be required in risk assessments based on *in vitro* data remain to be seen.

## Conclusion

The NRC vision for the future of toxicity testing suggests that PODs for risk assessments may be increasingly based on *in vitro* HTS data, a notion that has been incorporated into the U.S. EPA’s framework for the next generation of risk science. The technical definition of a POD derived from dose–response modeling has stimulated significant discussion within the current risk-assessment paradigm; the present study has extended this discussion to the case of HTS data using a large database comprising HTS experimental concentration–response curves generated during Tox21 Phase I. How the POD for HTS data should be designed to support future risk-assessment applications warrants further discussion. Although end point–specific definitions of the BMD, based on judgment applied on a case-by-case basis, are conceptually appropriate, they may be problematic in practice given the vast amount of data that will be generated through the greatly expanded application of robotically mediated high-throughput *in vitro* testing. Such rich data may require the use of standardized procedures and PODs for practical application and meaningful interpretation. The SNCD may provide a reference level that guides the determination of standardized BMDs, or similar potency-based measures, such that they are not subject to excessive uncertainty. Based on the present database, comprising > 8,000 HTS curves, such BMDs and BMDLs may need to be associated with a response higher than the standard responses of 5% or 10%. The SNCD may also be of potential use as a starting point for low-dose extrapolation in the process of establishing safe exposure limits.

## Supplemental Material

(157 KB) PDF
