# Supplementary material for: Comparison of Points of Departure for Health Risk Assessment Based on High-Throughput Screening Data
Source: Environ Health Perspect. 2016 Jul 6;125(4):623–33. doi: 10.1289/EHP408 (PMC5381992; doi:10.1289/EHP408)
Supplement: (157 KB) PDF [file EHP408.s001.acco.pdf]

Environ Health Perspect
DOI: 10.1289/EHP408

Note to readers with disabilities: *EHP* strives to ensure that all journal content is accessible to all readers. However, some figures and Supplemental Material published in *EHP* articles may not conform to 508 standards due to the complexity of the information being presented. If you need assistance accessing journal content, please contact ehp508@niehs.nih.gov. Our staff will work with you to assess and meet your accessibility needs within 3 working days.

Supplemental Material

Comparison of Points of Departure for Health Risk Assessment Based on High-Throughput Screening Data

Salomon Sand, Fred Parham, Christopher J. Portier, Raymond R. Tice, and Daniel Krewski

Table of Contents

1. Concentration-response modeling and estimation of PODs
2. Definition of the SNCD and the BMD
3. Analysis of NTP duplicates
   Table S1. Median effect at the SNCD (and the lower 5th and upper 95th percentiles) for NTP duplicates.
4. References

1. Concentration-response modeling and estimation of PODs

Concentration-response modeling was performed with the Hill model using a parametric bootstrap approach. The bootstrap approach was preferred over the profile likelihood method since initial analysis suggested that it was more suitable for derivation of different points of departure for a large number of curves in an automated manner. The profile likelihood method is programmatically more complicated as compared to the bootstrap approach, and it is also more difficult to generalize so as to handle the various concentration-response characteristics that may be present in a large database; the number of curves used as starting point in this study is ten times greater than that considered by Sand et al. (2011) [n = 11,240 in this study vs. n = 1,128 in Sand et al. 2011]. Implementation of the bootstrap approach is slower than the profile likelihood method; however, the processing time required for the profile likelihood method increases with the number of quantities (here, the number BMDs and SNCDs) that are derived from each curve, whereas the bootstrap method is practically independent of this. The approach for model fitting and derivation of PODs is described in detail below. Parts of the Matlab code can be made available upon request (salomon.sand@slv.se).

1. The Hill model, $\mu(d_i) = \alpha + \theta \frac{d_i^{\eta}}{\kappa^{\eta} + d_i^{\eta}}$, was fitted to a given curve assuming constant variance, resulting in estimates $\hat{\mu}(d)$ for the mean response, and an estimate, var for the variance of $\hat{\mu}(d)$. The Matlab function “lsqcurvefit” was used for model fitting. Curves that showed a non-significant concentration-response trend (according to a likelihood ratio test at significance level of p ? 0.05) after this initial step were not considered further. The constant variance assumption was evaluated under a separate analysis (data not shown). Briefly, the following relations were investigated using a linear model: 1) the

relation between concentration and the (absolute) residual associated with the model fitting, 2) the relation between log concentration and the (absolute) residual associated with the model fitting, and 3) the relation between the estimated response, $\hat{\mu}(d)$, and the (absolute) residual associated with the model fitting. The residuals did not systematically increase/decrease with concentration or response across the studied data sets (n? 10,000) (the distribution for the slope of the linear models was quite symmetrical with a mean/median close to zero) indicating that it was not necessary to assume non-constant variance, i.e. variance increasing with concentration or response. There are many possible models for concentration-response curves. We chose the Hill model for, among other reasons, consistency with earlier work with the SNCD approach (Sand et al., 2011). The Hill model is also one of the models that is recommended by EFSA (2009)

2. The likelihood estimator of the variance of the estimated mean response was adjusted to an unbiased estimator, $s^2 = var * m / (m - p)$, where m is the number of concentrations and $p = 4$ is the number of parameters for the mean response. The square root of s^2 was then multiplied by a correction factor (also applied in Sand et al. 2003)

$$k = \frac{\Gamma\left[\frac{m-p}{2}\right]}{\Gamma\left[\frac{m-p+1}{2}\right]} \times \frac{\sqrt{m-p}}{\sqrt{2}} \ ,$$

where *?* is the gamma function, resulting in *s*-hat, an unbiased estimate of *s*. For the number of concentration, *m*, in the range of 11 to 16, which is the case in the present study, the correction factor, *k*, is between 1.02 and 1.04. Thus, *s*-hat approximates to $\sqrt{s^2}$ in these analyses.

3. N = 1000 curves were then randomly generated from a normal distribution with standard deviation, s (s-hat, was used as input for the random number generator in Matlab) and means defined by $\hat{\mu}(d)$ corresponding to the applied concentrations (d) for the original curve.

4. The Hill model was fitted to each generated curve, and 10 different BMDs were estimated for each generated curve as described below.

    a. Extra effect: BMDs corresponding to extra effects of 5, 10, 20, 30, and 40% were estimated, where the extra effect was defined as a percent change in response relative to the estimated range of response (the difference between the maximum and minimum response value estimated by the Hill model fitted to generated curves). Using the Hill model above, the extra effect is $\frac{\mu(d)-\alpha}{\theta}$.

    b. Additional effect: BMDs corresponding to additional effects of 5, 10, 15, 20, and 25% were estimated, where the additional effect was defined as an absolute change in response compared to the estimated background response (estimated by the Hill model fitted to the generated curves). Using the Hill model above, the additional effect is $\mu(d) - \alpha$.

Two-sided 90% confidence intervals were established for each of the 10 BMDs based on the 1,000 samples generated; they correspond to the lower 5th and upper 95th percentiles

of the distributions comprising 1,000 BMD values. The point estimates of the different BMDs were based on the original model estimated in step 1.

5. A two-sided 90% confidence interval for the absolute effect was established over the experimental concentration range, based on the 1,000 generated concentration-response curves. SNCDs corresponding to signal-to-noise ratios (SNRs) of 1.0, 0.67, and 0.5 were then calculated, where the SNR is defined as

$$SNR = \frac{\hat{\mu}(d) - \hat{\mu}(0)}{U95 - L05}$$

where $\hat{\mu}(d) - \hat{\mu}(0)$ is the point estimate of additional effect at concentration, *d*, (based on the original model in step 1), and *U95* and *L05* denote the upper 95th and lower 5th confidence limits on the absolute effect at concentration, *d*. The SNR was computed at 20 points across the experimental concentration range, and the SNCDs are then solved as the doses corresponding to the three critical SNRs using spline interpolation.

6. Based on the 1,000 generated dose-response curves, a two-sided 90% confidence interval for (extra and additional) effect was derived at concentration corresponding to each of the three SNCDs (point estimates of the effects at the SNCDs were based on the original model in step 1).

7. The process described in step 1-6 was repeated for each of the 11,240 curves.

2. Definition of the SNCD and the BMD

The definition of the SNCD in the present study can be considered to be identical to that used in Sand et al. (2011), since the SNR was defined as the ratio between two absolute differences: $[\hat{\mu}(d) - \hat{\mu}(0)]/[U95 - L05]$. This was regarded as appropriate, since the concentration-response data used had been normalized. In the case of continuous data that is not normalized, the corresponding definition may have to be modified. For example, for continuous endpoints such as changes in body or organ weights assessment of relative/proportional changes in response has been suggested (Slob and Pieters 1998; Slob 2002), which is also reflected in the EFSA (2009) recommendation to define the BMD for continuous data as corresponding to a percent change in response relative to the background response. A definition of the SNR formulated on the relative scale is: $\left[\frac{\hat{\mu}(d)}{\hat{\mu}(0)}\right] / \left[\frac{U95}{L05}\right]$. Alternatively, the definition used in the present study might be applied after log-transformation of the response data.

The BMDs were derived under two different definitions, corresponding to extra and additional effects. As pointed out in the introduction, there are various BMD definitions available, particularly for continuous dose-response data, and some defaults have also been proposed. The extra effect definition corresponds to the extra risk definition that was previously used for the quantal response cancer bioassay data evaluated by Sand et al. (2011). Both extra effect and extra risk represent a response change in relation to the range of the concentration-response curve. The extra effect definition has been commonly used in applications of concentration-response modeling with in vitro data (Sand et al., 2012). The additional effect definition was also considered, since the normalization of the concentration-response data may be regarded to theoretically imply that the range of response should be 100 or -100. Thus, both definitions can

be considered to represent a response change in relation to the range of the response; in the case of extra effect the range of response is estimated by the model, and in the case of additional effect it is assumed that the range of response is 100 or -100 (although the fitted dose-response model is allowed to depart from this assumption). The relative effect (i.e., a percent change in response) that has been suggested as the default for BMD calculation with continuous data by the EFSA (2009), discussed above, is not regarded to be applicable to the present type of data, since it has been normalized, and may include both positive and negative response values. The extra/additional risk/effect definitions, or extensions thereof, might be more generally applied across various data types.

3. Analysis of NTP duplicates

Summary results of the effect at the SNCD for NTP duplicates are shown in Supplemental Material, Table S1. This analysis has been performed in a manner identical to that for the whole database comprising n = 8,456 included curves. The median effects, with 90% confidence intervals, at the SNCD for the case of separate analysis of duplicates are very similar to those associated with the SNCD from the analysis of merged duplicates. The median effects at the SNCD are also similar to those for the whole database comprising n = 8,456 curves (Table S1 and Figures 6 and 7 in the main article).

Table S1. Median effect at the SNCD (and the lower 5th and upper 95th percentiles) for NTP duplicates.

Effect Definition	Effect Type	Quantity	Separate Analysis	Merged Analysis
Extra effect	Upper 95th confidence bound	$SNCD_{1.0}$	0.36 (0.07 - 0.94)	0.37 (0.09 - 0.94)
		$SNCD_{0.67}$	0.23 (0.04 - 0.77)	0.23 (0.05 - 0.75)
		$SNCD_{0.5}$	0.16 (0.03 - 0.65)	0.17 (0.03 - 0.54)
	Point estimate	$SNCD_{1.0}$	0.22 (0.05 - 0.52)	0.21 (0.06 - 0.51)
		$SNCD_{0.67}$	0.11 (0.03 - 0.33)	0.12 (0.03 - 0.31)
		$SNCD_{0.5}$	0.07 (0.02 - 0.22)	0.07 (0.02 - 0.22)
Additional effect	Upper 95th confidence bound	$SNCD_{1.0}$	25 (6.9 - 59)	24 (7.4 - 57)
		$SNCD_{0.67}$	17 (3.8 - 45)	17 (4.0 - 45)
		$SNCD_{0.5}$	13 (2.8 - 38)	13 (2.9 - 39)
	Point estimate	$SNCD_{1.0}$	15 (4.3 - 38)	15 (4.6 - 36)
		$SNCD_{0.67}$	8.2 (2.1 - 22)	8.0 (2.2 - 20)
		$SNCD_{0.5}$	5.3 (1.5 - 15)	5.2 (1.5 - 14)

Note: The results for separate analysis of duplicates is based on 536 curves (N = 268 duplicates), and the results for merged analysis of duplicates is based on N = 271 merged duplicates. The reason for the discrepancy in the number of duplicates (268 vs. 271) is due to the fact that four additional duplicates had significant concentration-response trends when merged in the merged analysis according to criteria described in Supplemental Material, Section 1. There were a total of 320 NTP duplicates with curves in classes 1 and 2 (see Materials and Methods in the main article regarding definition of curve classes). This was reduced to 268 and 271 curves, respectively, since exclusion criteria identical to that used in the analysis of the whole database (n = 8,456) was also applied in this analysis; see the section on Comparison of PODs in the Material and Methods in the main article.

4. References

EFSA (European Food Safety Authority). 2009. Guidance of the Scientific Committee on a request from EFSA on the use of the benchmark dose approach in risk assessment. The EFSA Journal 1150:1-72.

Sand S, von Rosen D, Falk Filipsson A. 2003. Benchmark calculations in risk assessment using continuous dose-response information: the influence of variance and the determination of a cut-off value. Risk Anal 23: 1059-1068.

Sand S, Portier CJ, Krewski D. 2011. A signal-to-noise crossover dose as the point of departure for health risk assessment. Environ Health Perspect 119:1766-1774.

Slob W, and Pieters MN. 1998. A probabilistic approach for deriving acceptable human intake limits and human health risks from toxicological studies: general framework. Risk Anal 18:787-798.

Slob W. 2002. Dose-Response Modeling of Continuous Endpoints. Toxicol Sci 66: 298-312.
